# Deoxynivalenol Degradation by Various Microbial Communities and Its Impacts on Different Bacterial Flora

**DOI:** 10.3390/toxins14080537

**Published:** 2022-08-05

**Authors:** Chenggang Cai, Miaomiao Zhao, Feng Yao, Ruiyu Zhu, Haiying Cai, Suqin Shao, Xiu-Zhen Li, Ting Zhou

**Affiliations:** 1 College of Biological and Chemical Engineering, Zhejiang University of Science and Technology, Hangzhou 310023, China; 2 Guelph Research and Development Centre, Agriculture and Agri-Food Canada, Guelph, ON N1G 5C9, Canada

**Keywords:** deoxynivalenol, microbial detoxification, microbial diversity, high-throughput sequencing

## Abstract

Deoxynivalenol, a mycotoxin that may present in almost all cereal products, can cause huge economic losses in the agriculture industry and seriously endanger food safety and human health. Microbial detoxifications using microbial consortia may provide a safe and effective strategy for DON mitigation. In order to study the interactions involving DON degradation and change in microbial flora, four samples from different natural niches, including a chicken stable (expJ), a sheep stable (expY), a wheat field (expT) and a horse stable (expM) were collected and reacted with purified DON. After being co-incubated at 30 °C with 130 rpm shaking for 96 h, DON was reduced by 74.5%, 43.0%, 46.7%, and 86.0% by expJ, expY, expT, and expM, respectively. After DON (0.8 mL of 100 μg/mL) was co-cultivated with 0.2 mL of the supernatant of each sample (i.e., suspensions of microbial communities) at 30 °C for 96 h, DON was reduced by 98.9%, 99.8%, 79.5%, and 78.9% in expJ, expY, expT, and expM, respectively, and was completely degraded after 8 days by all samples except of expM. DON was confirmed being transformed into de-epoxy DON (DOM-1) by the microbial community of expM. The bacterial flora of the samples was compared through 16S rDNA flux sequencing pre- and post the addition of DON. The results indicated that the diversities of bacterial flora were affected by DON. After DON treatment, the most abundant bacteria belong to *Galbibacter* (16.1%) and *Pedobacter* (8.2%) in expJ; *Flavobacterium* (5.9%) and *Pedobacter* (5.5%) in expY; *f_Microscillaceae* (13.5%), B1-7BS (13.4%), and RB41 (10.5%) in expT; and *Acinetobacter* (24.1%), *Massilia* (8.8%), and *Arthrobacter* (7.6%) in expM. This first study on the interactions between DON and natural microbial flora provides useful information and a methodology for further development of microbial consortia for mycotoxin detoxifications.

## 1. Introduction

Deoxynivalenol (DON) is a secondary metabolite mainly produced on corn, wheat, and other cereal crops by several Fusarium species, including *F. graminearum* and *F. avenaceum* [1,2]. A warm and wet environment will aggravate DON production and its accumulation in grains [3]; then, DON enters the food chain [4,5], resulting in serious toxic effects on humans and animals [6,7,8]. The highest detected DON content was 41,157 μg/kg in a wheat sample, which was one of the 96% contaminated wheat samples detected [9,10]. It is estimated that more than one billion people worldwide, especially those in developing countries [11,12], are directly and indirectly exposed to high concentrations of DON and its metabolites due to fungal contamination of food and feed. Low-dose ingestion of DON may damage the gut [13], cause diarrhea, inhibit nutrient absorption, and lead to weight loss [14]; DON intakes at high doses can cause vomiting and anorexia and impairment of the hematopoietic system and immune barrier function [15,16].

If DON level is too high in the grains, the grain will lose its economic value as food or feed. One of the efficient ways to utilize the contaminated grain is by composting, a biological method to degrade DON, in addition to other physical and chemical methods. Biotransformation reactions, including acetylation, glycosylation, ring cleavage, hydrolysis, deamination, and decarboxylation, have been studied as biological approaches for DON mitigation [17] and are considered safe, effective, and economical methods [18]. Some purified microbes have been reported to pose DON degradation functionality, including the bacteria *Slackia* sp., which de-epoxidized DON to nontoxic DOM-1 [19]; the culture supernatant of heat-killed *Lactobacillus plantarum* could effectively reduce the toxicity of DON in porcine intestinal tissue [20]; *Aspergillus* NJA-1 isolated from soil had a degradation rate of 94.4% after being cultured with DON for two weeks [21]; and *Devosia* mutans 17-2-E-8 has been extensively studied for its conversion of DON to 3-keto-DON and then 3-epi-DON by a two-step enzymatic system [22,23].

In addition to screening for single strains, DON-detoxifying microbial consortia have also been acquired for mitigating DON-contaminated grains and feeds. For example, a consortium of enhanced microorganisms originated from soil samples collected from farmlands in Canada was able to transform DON into de-epoxy DON completely [24]. Mixed microorganisms have also been reported to convert DON to 3-keto-DON [25].

Although it is necessary to isolate a single microbe for the feed and food industries and for further biological study, it is usually time-consuming for the isolation of a single microorganism. This effective microorganism (EM) concept had been reported for many years in agricultural crops cultivation. However, it is hypothesized that the microbial community with more degradation effects may have better practical utilization potential for the fertilizer or environment purpose. Therefore, we have focused on the group microorganisms (GM) concept in DON degradation studies for several years; for example, over ten kinds of organic fertilizer possessing the group microorganisms (GM) have been tested to degrade DON in our previous studies; DON was almost 100% degraded (data not published). Based on the fact that bacterial flora from various sources involving soil and animal guts have the DON degradation ability, and that there are few reports on the interaction influences of the microbial flora on DON and vice versa, the aim of this study was to investigate the DON reduction effects and product formation by four typical samples from a chicken stable (expJ), a sheep stable (expY), a wheat field (expT) and a horse stable (expM), which provides useful information on potential microbial sources for DON degradations or detoxifications. The comparisons of the bacterial diversities of the samples before and after DON treatment lead to understanding how DON may affect microbial flora, paving ways for identifying DON detoxification microbial species or consortia, particularly using genomics approaches. Additionally, the results will provide the GM for further studies on the culture medium optimization, interactions between different kinds of microbes, DON degradation mechanisms and so on in the future. The high-throughput sequencing analysis in this study also provided an effective method to isolate the promising single strains for high-density cultivation and inoculation for DON contaminated cereals.

## 2. Results

### 2.1. Degradation of DON by Different Natural Microbial Communities

**Figure 1:**
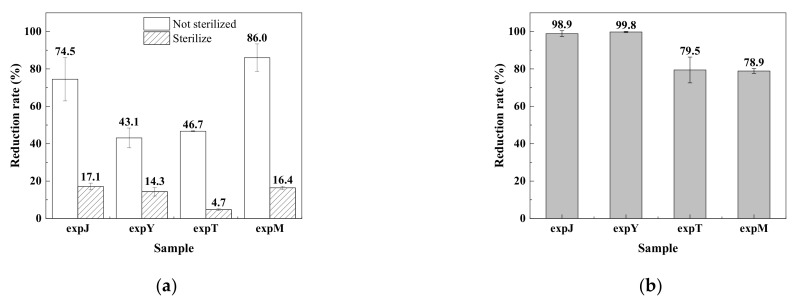
The reduction rate of DON in different samples before and after sterilization at 96 h (**a**); the reduction rate of DON in the supernatant of different samples after DON treatment and was re-incubated with DON solution for 8 days (**b**).

Four microbial sources representing four typical farm microbial sources were used in this study, i.e., the microbes in three representative animal stables (chicken, sheep and horse, the manure from which are usually composted for using as fertilizer) and a natural farm field (wheat field). After purified DON was incubated for 96 h with microbial communities of four different sources, sampled from the chicken stable (expJ), the sheep stable (expY), the wheat field (expT) and the horse stable (expM), respectively, DON level was reduced by all the microbial communities. The microbial suspension of expM showed the greatest reduction, reducing DON by 86.0%, followed by expJ by 74.5%, expT by 46.7%, and expY by 43.1% (Figure 1a). When DON was added into each of the four sterilized samples to determine possible absorption effects by soil particles, it was found that DON concentration decreased slightly in all samples, including expJ by 17.1%, expY by 14.3%, expT by 4.7%, and expM by 16.4% (Figure 1a). Therefore, the adjusted DON degradation rates of the four microbial samples were 57.4% (expJ), 28.8% (expY), 42.0% (expT) and 69.6% (expM), respectively, which resulted from the activities of the microbial communities. Both adsorption and degradation effects of the microbes were further studied using the co-cultivation method to compare the combinations of the supernatant vs. the sterilized supernatant and with or without DON. After 96 h of static cultivation, DON in the sterilized supernatants of all samples kept at a primary constant concentration, i.e., no reduction; however, DON levels in the unsterilized supernatants of the four samples reduced very significantly, by 98.9% (expJ), 99.8% (expY), 79.5% (expT) and 78.9% (expM), respectively (Figure 1b), indicating that DON reduction was mainly due to microbial degradation rather than absorption.

**Figure 2:**
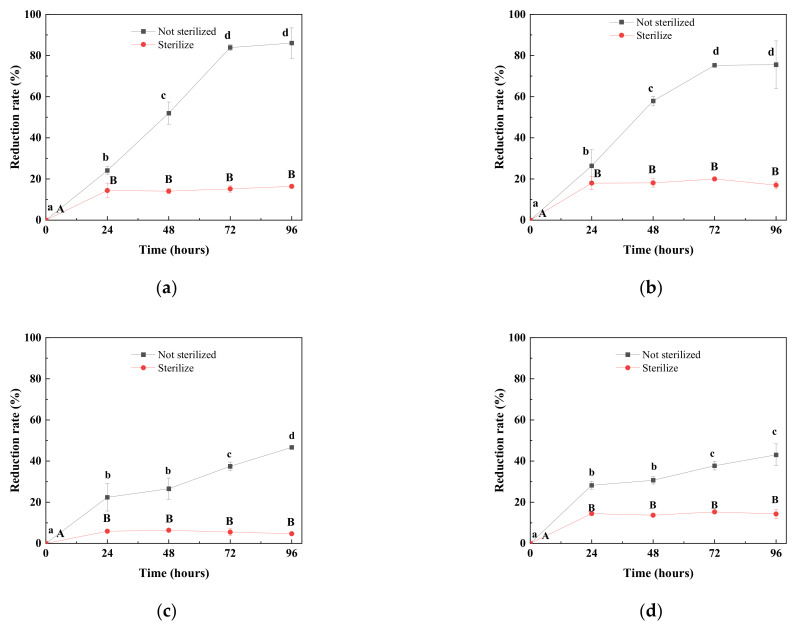
Degradation rate of DON by expM (**a**), expJ (**b**), expT (**c**), and expY (**d**) flora during 96 h incubation (*p* < 0.05). Different letters of a, b, c, d and A, B indicates significantly differences of the results (*p* < 0.05).

The microbial communities of expM (a), expJ (b), expT (c) and expY (d) were further analyzed for time-course DON degradation, and the results are shown in Figure 2. The DON levels in the sterilized samples were almost unchanged after 24 h, indicating the DON adsorption capacity by the samples had reached maximum within 24 h. The results showed that after cultivation for 72 h, the DON degradation rate remained at high levels in the unsterilized expM (a) and expJ (b), and showed no significant difference at 96 h. The degradation rates in the unsterilized expT (c) and expY continued to increase until 96 h and were lower than other two groups.

**Figure 3:**
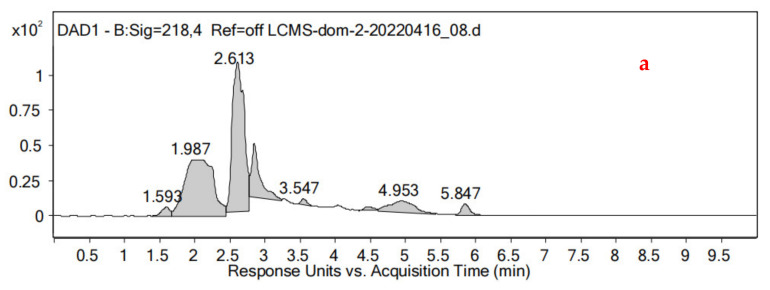

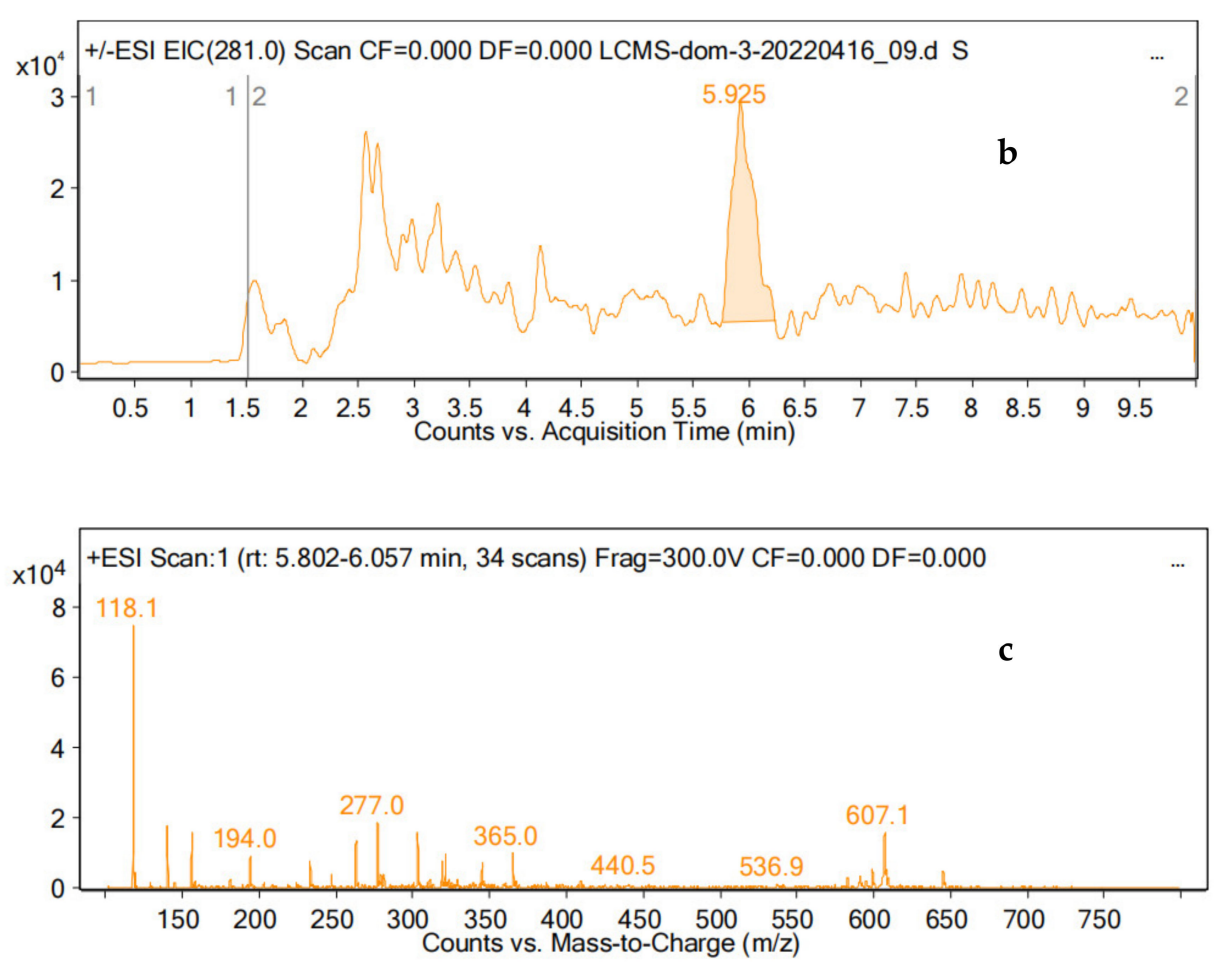
UPLC-MS chromatogram of DON reduction product in expM cultivation sample. The UPLC analysis was performed on a Zorbax Eclipse XDB-C18 column (**a**), a SIM scan of the ion at *m*/*z* 281 leads to a peak at t_R_ 5.925 min (**b**), corresponding to the component of DOM-1 (fw = 280) (**c**).

**Figure 4:**
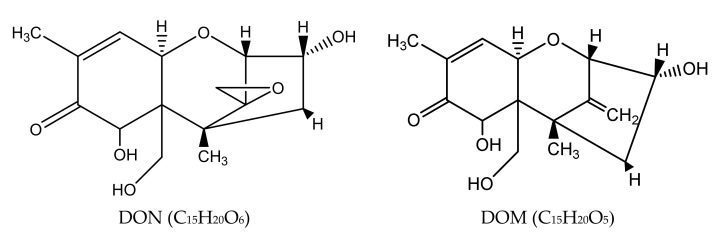
Molecular structure of DON and DOM.

The DON reduction products in expJ, expY and expT samples were not detected, and DON was completely degraded. The limit of detection (LOD) and limit of quantification (LOQ) of DON were of 0.05 ppm and 0.15 ppm, respectively. A new peak was found in the HPLC (high-performance liquid chromatography) chromatogram (Figure 3) in the expM sample; the peak was further analyzed by an ultra-performance liquid chromatography–mass spectrometry (UPLC-MS), and it was found to be de-epoxy DON (DOM-1) (Figure 4).

### 2.2. The Diversities of Bacterial Flora in the DON-Degrading Samples

#### 2.2.1. OTU Cluster Analysis

**Figure 5:**
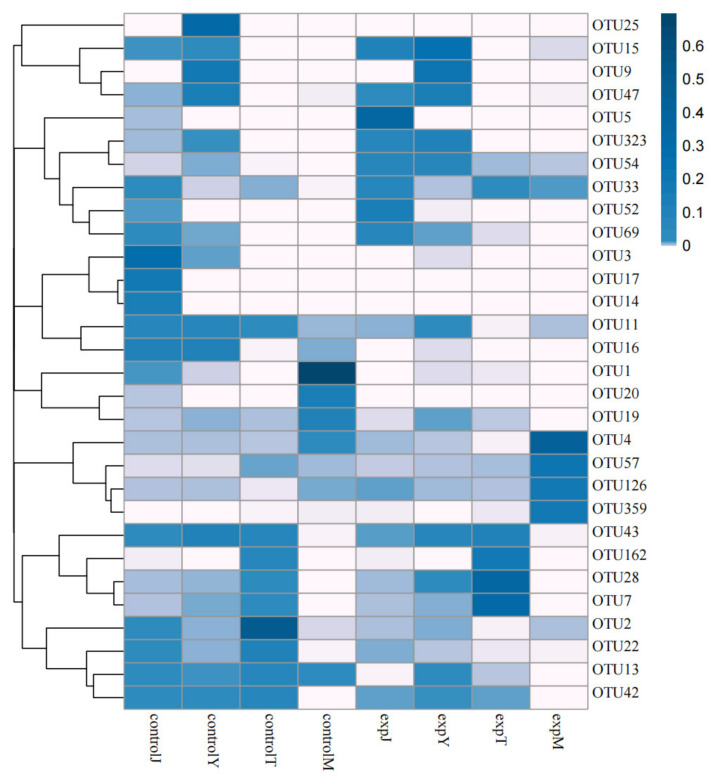
Heat map of OTU abundance clustering.

**Figure 6:**
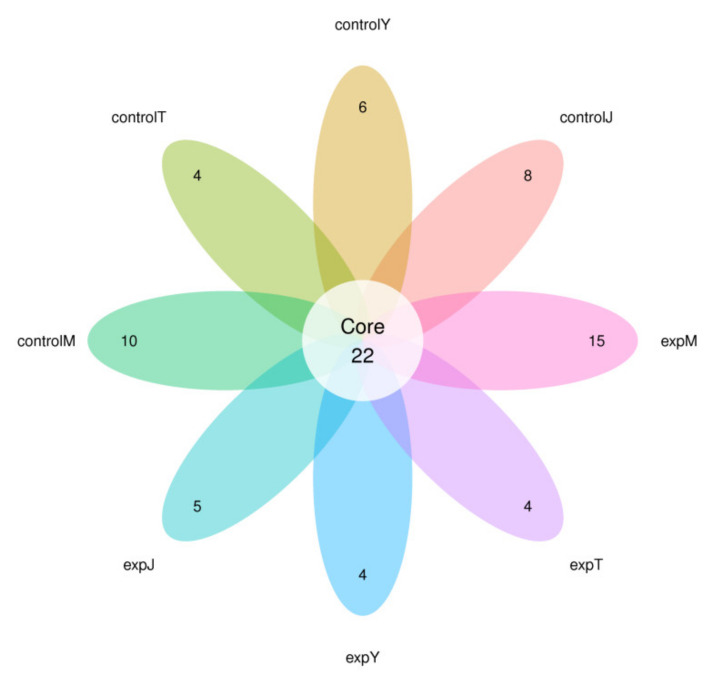
Venn diagram of different OTUs. 4, 5, 6, 8, 10, 15 were number of OTUs.

According to previous studies showing that most of the single strains showing DON degrading capabilities were of bacteria, high-throughput sequencing of the bacteria in the samples was performed in this study. The DON-treated samples (30 °C, 130 rpm, 96 h) of expJ, expY, expT, and expM, as well as their control groups (without DON; 30 °C, 130 rpm, 96 h) of controlJ, controlY, controlT, and controlM were subjected to microbial-diversity analysis using the Anshengda Illumina MiSeq/NovaSeq sequencing platform for sequencing 16S rDNA amplicons. In order to understand the number of species, genera, and other information in the sequencing results of mixed bacterial species, the sequencing operation was performed, and all the sequences were classified into multiple groups according to their similarity to each other. Each group was defined as an OTU, and 97% of the sequences were divided into OTUs at a similar level; bioinformatics statistical analysis was performed to obtain the species distribution abundance information of each sample, and the 30 OTUs with the highest default abundance were plotted (Figure 5). Compared with the control groups, the abundances of OTU4, OTU33, and OTU359 of the expM flora; OTU28 and OTU7 of the expT flora; OTU5, OTU323, and OTU54 of the expJ flora; and OTU28 and OTU15 of the expY flora after DON treatment were significantly increased. According to the results of the OTUs’ cluster analysis, the number of OTUs that were common and unique to the above eight bacterial groups was counted, and the OTU Venn diagram was drawn (Figure 6). There were 22 OTUs shared in the eight bacterial groups, and the number of OTUs unique to controlM, controlT, controlY, controlJ, expM, expT, expY, and expJ were 10, 4, 6, 8, 15, 4, and 5, respectively.

**Figure 7:**
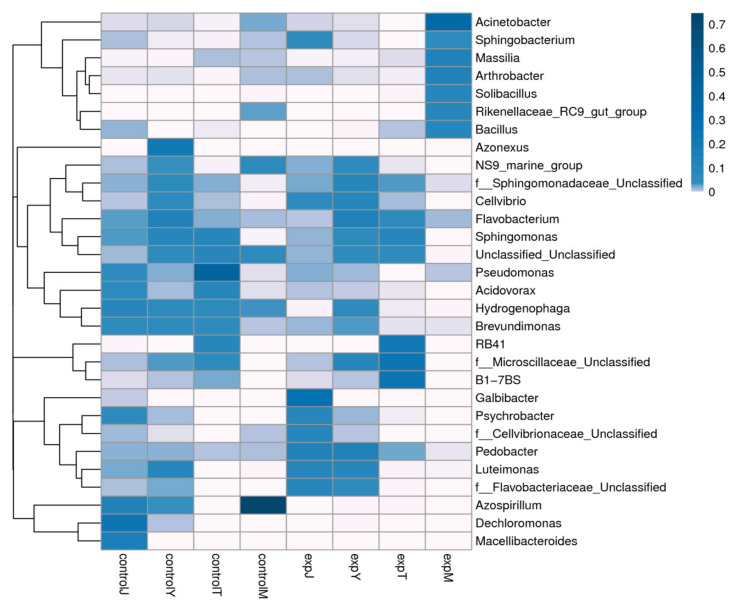
Species distribution heat map.

In order to obtain the species classification information corresponding to the OTU, a representative sequence was selected for each OTU, and the representative sequence was annotated by species classification using the RDP classifier to obtain the community composition of each sample. The sample results were drawn into a heat map based on the distribution of TOP30 species at different genus levels (Figure 7). Compared with the control group, *Galbibacter*, *Sphingobacterium*, *f_Flavobacteriaceae*, *Cellvibrio*, *f_Cellvibrionaceae*, *Pedobacter*, and *Luteimonas* in the expJ flora increased significantly after DON treatment; *Luteimonas*, *f_Flavobacteriaceae*, and *Pedobacter* in the expY flora increased significantly; RB41, B1-7BS, and *f_Microscillaceae* increased significantly in the expT flora; and *Acinetobacter*, *Sphingobacterium*, *Masilia*, *Arthrobacter*, *Solibacillus*, *Rikenellaceae* RC9 gut group, and *Bacillus* increased significantly in the expM flora.

**Figure 8:**
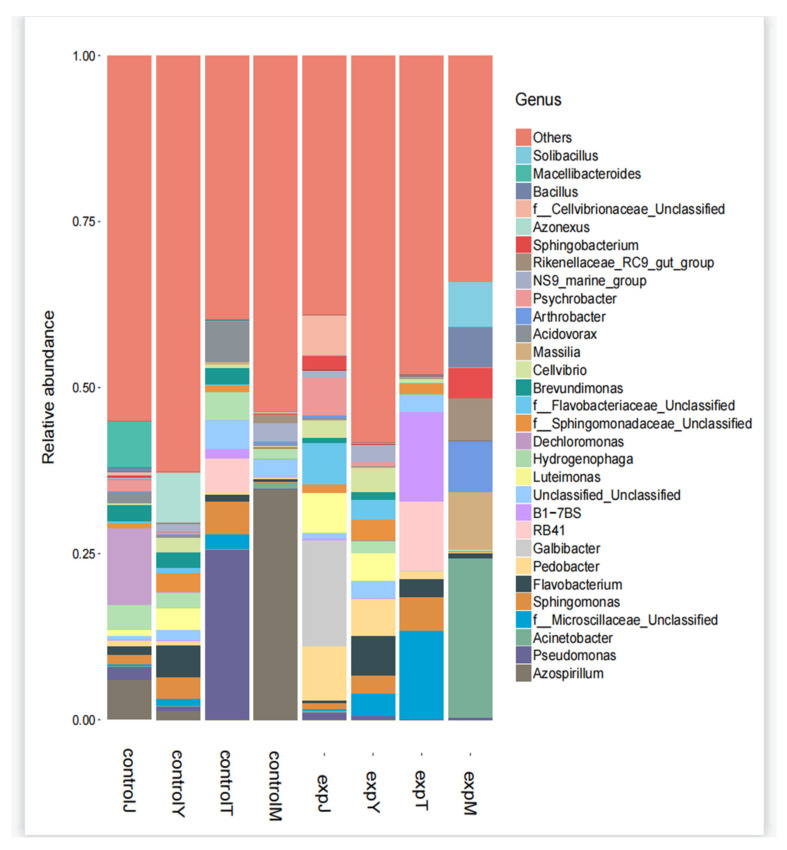
Species distribution histogram.

There were 177 genera in controlJ, 146 genera in expJ, 179 genera in controlY, 162 genera in expY, 142 genera in controlT, 143 genera in expT, 117 genera in controlM, and 72 genera in expM. Based on the high-throughput sequencing results, the distribution of the Top 30 species at different genus levels for each sample was drawn as a histogram (Figure 8). The controlJ flora mainly included *Dechloromonas*, *Macellibacteroides*, and *Azospirillum*, accounting for 11.7%, 7.0%, and 6.1%, respectively; the expJ flora mainly included *Galbibacter* and *Pedobacter*, accounting for 16.1% and 8.2%, respectively. The controlY flora mainly included the *Azonexus* and *Flavobacterium* genera, accounting for 7.6% and 4.8%, respectively; and the expY flora mainly included *Pedobacter* and *Flavobacterium* genera, accounting for 5.5% and 5.9%, respectively. The controlT flora mainly included *Pseudomonas*, *Acidovorax*, and RB41 genera, accounting for 25.7%, 6.1%, and 5.1%, respectively; the expT flora mainly included f_*Microscillaceae*, B1-7BS, and RB41, accounting for 13.5%, 13.4%, and 10.5%, respectively. The controlM flora mainly included *Azospirillum*, accounting for 34.7%, and the expM flora mainly included the genera *Acinetobacter*, *Massilia*, and *Arthrobacter*, accounting for 24.1%, 8.8%, and 7.6%, respectively. It can be seen from the above data that compared with the flora of the control group, the genus of the flora after DON treatment changed greatly, indicating that DON had an impact on the flora.

**Figure 9:**
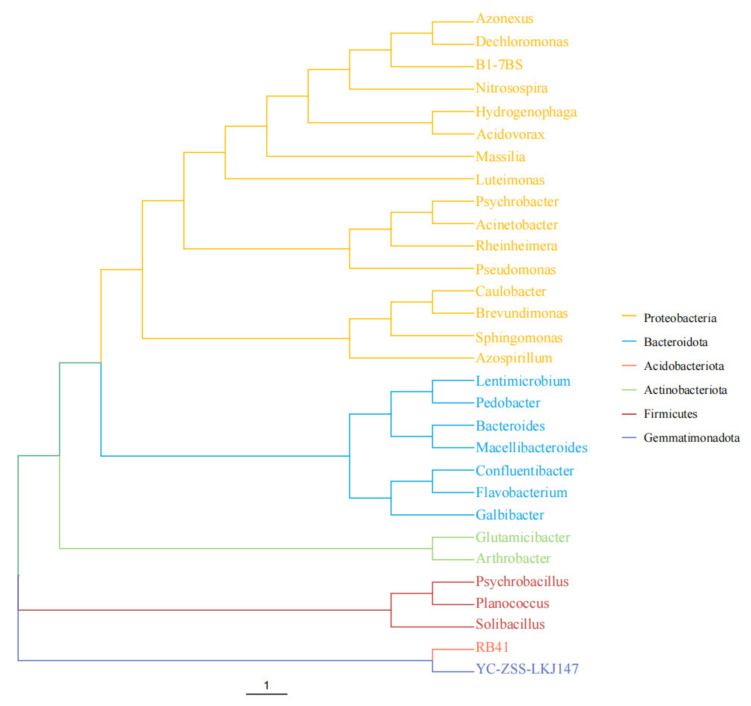
Horizontal evolutionary tree of Top 30 genera. “1” is the unit length of the difference value between different organisms.

For the genus-level OTU sequences of the Top 30, a phylogenetic tree was constructed according to the maximum likelihood method (Figure 9). The results showed that the mixed flora of the samples was mainly composed of six phyla of *Proteobacteria*, *Bacteroidota*, *Acidobacteriota*, *Actinobacteriota*, *Firmicutes*, and *Gemmatimonadota*.

#### 2.2.2. Alpha Diversity Analysis

**Figure 10:**
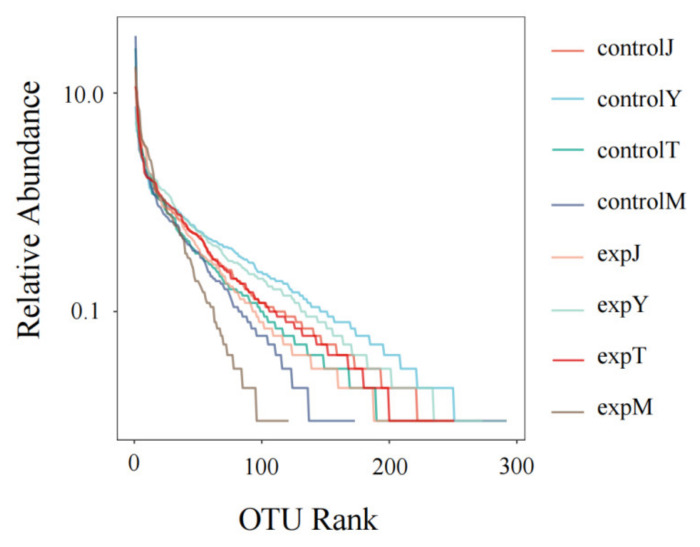
Rank–Abundance Curve.

**Figure 11:**
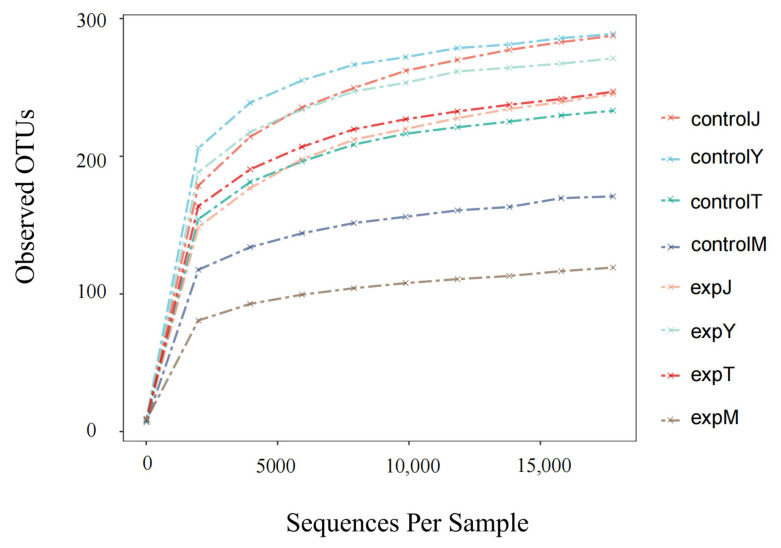
Dilution Curve.

In community ecology, alpha diversity mainly focuses on the diversity analysis of a single sample, which can reflect the number of species in the microbial community and estimate the species abundance and diversity of the environmental community through a series of statistical index analyses. The alpha diversity index of the flora of the eight samples is shown in Table 1. The ace index and the Chao1 index of the flora after DON treatment were all lower than those of the control group except for expT, and the total number of species was significantly reduced; the Shannon index and Simpson index of the expT and expM flora exponential after DON treatment were increased compared with the control group, indicating an increase in biodiversity, with a decrease in expJ and expY biodiversity. According to the rank–abundance curve (Figure 10), there was no significant difference in species abundance between the controlT and expT, and the species abundance of the rest of the DON-treated flora decreased compared with the control group. As shown in the dilution curve (Figure 11), with the increase in the number of sequences, the OTUs of each sample also increased and eventually became flat, and only a small amount of OTUs could be obtained by continuing sampling. At this time, the sample size was sufficient. The most abundant samples were close to 300.

#### 2.2.3. Beta Diversity Analysis

**Figure 12:**
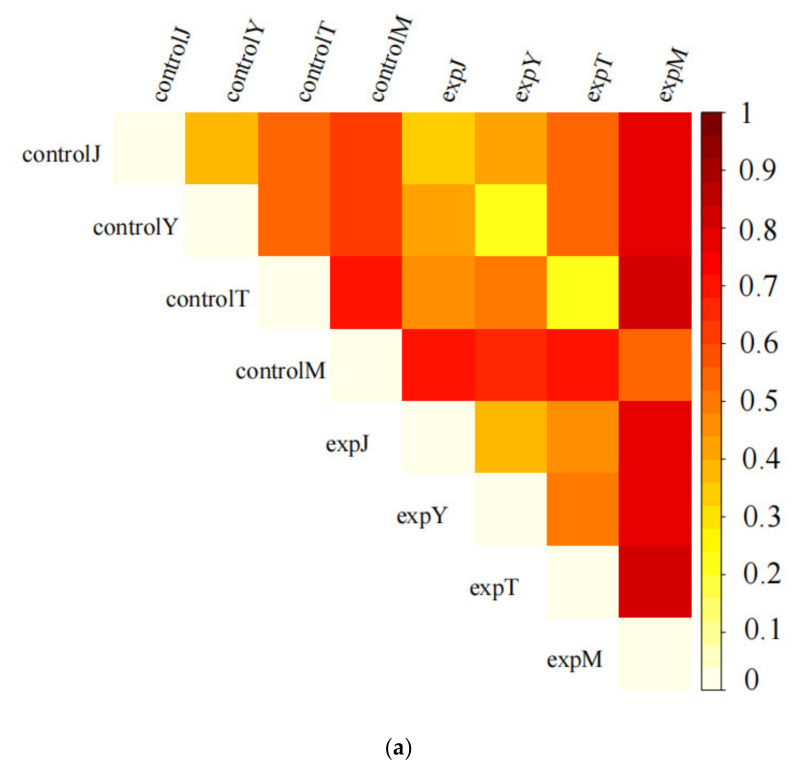

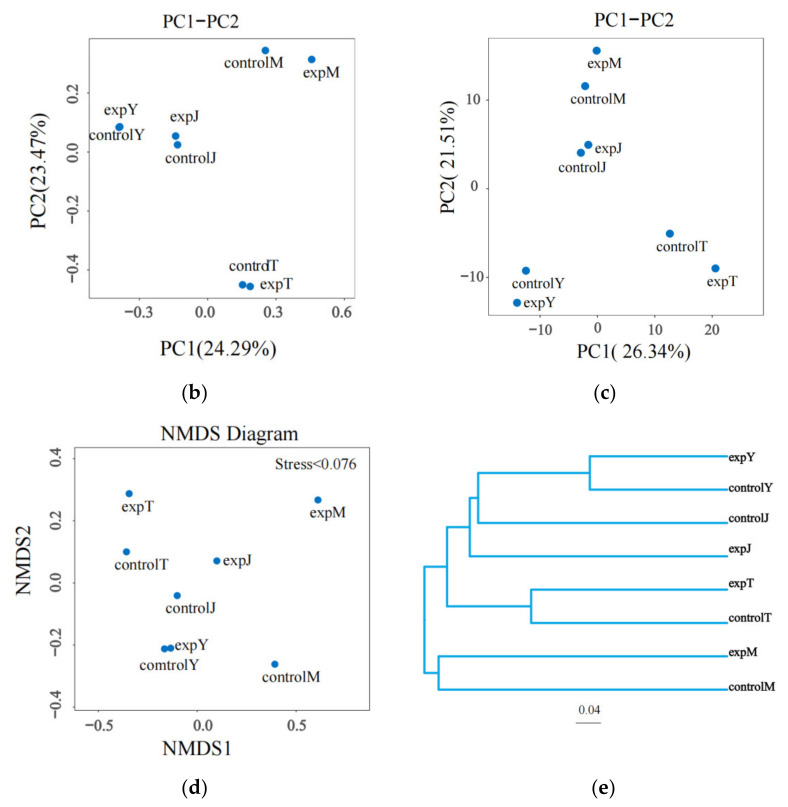
Cluster analysis based on the UniFrac (**a**), PCoA (**b**), PCA (**c**), NMDS (**d**), and UPGMA (**e**)-Tree methods, (**a**) is the distance matrix heat map, the color indicated the dissimilarity coefficient between two samples, which reflects the difference in diversity between different samples; (**b**) presented the visual coordinates of the similarity or difference of different groups; (**c**) indicated the dissimilarity coefficient of the samples as principal components; (**d**) indicated the sample similarity distance matrix for dimension reduction ranking analysis on the two-dimensional plane, the distance between sample points in the same group of UPGMA tree plot indicated the repeatability of the sample, and the distance between samples in different groups reflected the difference in the rank (data ranking) of the sample distance between groups; (**e**) indicated control and treatment groups clustered significantly among the groups in the UPGMA-Tree plot.

The β-diversity value is the dissimilarity coefficient between two samples, which reflects the difference in diversity between different samples. The distance between samples is calculated by using the evolution and abundance information between each sample sequence to reflect whether there is a significant microbial community shared between the samples. Differences, in order to avoid errors, use a variety of analytical methods for evaluation. The samples were processed by the UniFrac distance matrix, Principal Co-ordinates Analysis (PCoA), Principal Components Analysis (PCA), Non-metric multidimensional scaling (NMDS) analysis, Unweighted PairGroup Method with Arithmetic Mean (UPGMA)-Tree cluster analysis, and other methods. As shown in the distance matrix heat map (Figure 12a), the dissimilarity coefficient between expJ and controlJ was less than 0.4; the dissimilarity coefficient between expY and controlY, expT, and controlT was less than 0.3; and the dissimilarity coefficient between expM and controlM was less than 0.6. As shown in the PCoA diagram (Figure 12b), PCA diagram (Figure 12c), and NMDS diagram (Figure 12d), the distance between the sample points of the flora after DON treatment and the sample points of the control group was short, and the community similarity was high. As shown in the UPGMA-Tree plot (Figure 12e), the control and treatment groups clustered significantly among the groups.

## 3. Discussion

Microbial detoxifications have shown great potential in DON mitigations. DON was completely degraded when the microbes consumed DON as a carbon source [26], and the products usually showed low toxicity, such as DOM-1. There are diverse microorganisms in soils, plant debris, animal guts and animal excrements [27], which serve as reservoirs for microbes with unique functionalities [28]. Studies have reported that rumen microbiota and certain bacteria from chicken guts are capable of transforming DON [25,26]; DON-detoxifying microorganisms have also been obtained from various soils and plant surfaces [21,24,25,29,30,31,32,33]. In this study, the four samples were collected from very different environments, including chicken stable (expJ), sheep stable (expY) and horse stable (expM) as well as wheat field (expT), but all showed DON degradation activities, indicating widespread microorganisms with DON detoxification functionality, and also implying a commonness of DON presence in diverse natural niches. However, there is almost no knowledge on the interactions between DON and natural microbial communities. This high-throughput study on the changes in bacterial flora aims to determine primarily whether and how DON may affect natural microbial communities and explore effective strategies and methodologies for such studies. The vast majority of the bacterial populations in the four greatly varied samples were detected by 16S rDNA sequence analysis, which can reflect the diversity of the microbial communities and their interactions with DON.

Biological detoxification is the microbial and enzymatic conversions or transformations of mycotoxins, e.g., DON, into other low-toxicity or non-toxic substances. Several DON-transformed products or intermediates including DOM-1 [19], de-epoxy DON [24] and 3-keto-DON [25] have been reported. The *Devosia* mutans 17-2-E-8 has been extensively studied for its conversion of DON to 3-keto-DON and then 3-epi-DON by a two-step enzymatic system [22,23]. *Devosia* mutans 17-2-E-8, isolated from soil [31], metabolizes DON to the much less toxic 3-epi-DON [23,34]. *Nocardioides* sp. strain WSN05-2, identified from the soil of wheat fields, was able to convert 1000 μg/mL DON to 3-epi-DON [35]. *Bacillus* sp. LS100 originated from chicken intestine showed very effective activities and was able to completely transform DON to the less toxic DOM-1 [29]. Although there have been some successful cases of showing single microbial strains with DON detoxification capabilities similar to the above-mentioned examples, the acquisition of a single purified isolate with DON reduction abilities has been challenging and very time-consuming, which has been experienced by many researchers worldwide. On the other hand, there may be advantages in using active microbial communities or consortia for DON detoxification, such as higher degradation efficiencies, more tolerant cultivation conditions and better potential for environmental applications; a research report by the Prairie Agricultural Machinery Institute (PAMI) also recommended the composting method as the most simple and effective process to dispose of *Fusarium*-damaged wheat [36], which utilizes microbial communities (mainly from animal digest) as the group microorganisms to digest the DON-contaminated wheat crops.

Another study focused on ochratoxin A (OTA) degradation using Tibetan kefir grains (TKG) as a starter was carried out and the results indicated that *Lactobacillus kefiranofaciens* and *Kazachstania turicensis* were the most abundant bacterial and fungal taxa in TKG; TKG removed more than 90% of the ochratoxin A (OTA) after 24 h fermentation, and *Kazachstania unisporus* AC-2 exhibited the highest removal capacity (~46.1%) [37].

There are many uncultivable microbes presented in soil, animal guts and other niches; mixed microbes have shown great degradation abilities for natural substances including DON and its derivatives. Practically, some of the heavily infected wheat crops would not be harvested, resulting in high DON levels in the soil; the naturally occurred DON residues in the wheat field might be degraded by the microbes in the soil. The composting process is another example of the utilization of a microbial community to degrade DON. There have been studies on the effects of microbial flora on DON degradation; Islam et al. screened a microbial community capable of converting DON to DOM-1 from 150 collected soil samples, and a 16S rRNA gene sequence analysis indicated the existence of at least six genera, including *Serratia*, *Clostridium*, *Citrobacter*, *Enterococcus*, *Stenotrophomonas*, and *Streptomyces* [24], and 11 trichothecenes were converted to a less toxic, de-epoxidized form by a soil microbial consortium [38]. Wilson et al. reported screening plant and soil samples for microorganisms capable of degrading trisporene and, ultimately, identified two mixed cultures that consistently reduced DON levels through oxidation to 3-keto-DON [39]. Through high-throughput sequencing, it was found that the four DON-treated samples in the study contained bacteria of *Devosia*, *Nocardioides* and *Bacillus*, and their degradation products included 3-keto-DON, 3-epi-DON and DOM-1 [22,29,35]. Another high-throughput sequencing analysis of vomitoxin-degrading bacteria from soil [26] showed that the bacterial consortium LG-6 with DON-degrading capacity was obtained from soil, *seudochrobactrum*, *Pseudomonas*, *Delftia*, *Devosia*, and *Achromobacter* were further tested through the dilution method, and *Pseudomonas*, *Devosia* and *Delftia* are essential for the degradation of DON.

There is relatively steady microbial flora at different areas and special locations. The microbial species vary at different areas and change with environmental conditions; therefore, it is important to compare the various samples from the four different locations or other areas at different time or environmental conditions. The four selected samples in this study were chosen on the fact that the microbes from the soil and animal guts/waste generally contain DON degradation bacteria and, therefore, there are better opportunities to isolate the potential DON-degrading bacteria from those samples. The high-throughput sequencing analysis was one of the effective methods to narrow the potential promising microbes with DON degradation capability. Although more detailed studies are necessary, the results from the current study will provide some useful information for further single or group strains isolation and application. In view of the fact that the reported single DON degradation strains are mainly bacteria, the bacterial 16S rDNA high-throughput sequencing analysis was performed in this study. The bacterial flora, the species and diversity of the bacteria were determined. It was found that DON could affect the microbial diversity, but the effect could vary greatly for different microbial communities. This is understandable as there are bacterial species/strains variations among those microbial communities from different natural niches; different bacteria may respond to DON very differently, either being inhibited or enhanced. In this study, the microbial communities with reduced bacterial diversity by DON, e.g., expJ and expY, were more effective on DON degradation. It is possible that during the co-cultivation of DON and the sample supernatants, i.e., mixed microbial suspensions, the bacterial species being enhanced by DON might predominate or inhibit the growth of certain other bacteria, resulting in reduced microbial diversity.

It is the first time the interactions between DON with the microbial flora in a wheat field and three different animal stables were studied. The microbial flora from dissimilar sources containing very different microbes showed a high DON degradation rate. The use of microbial communities to transform mycotoxins in food or feed has great industrial application potential, especially for those heavy DON-contaminated cases under uncontrolled conditions. More intensive studies to identify the microorganism(s) responsible for the DON degradations in each of the varied samples should pave the bases for the development of effective strategies to mitigate DON in different agricultural and environmental systems.

## 4. Materials and Methods

### 4.1. Samples and Chemicals

All samples were collected between September and October 2021; the samples were transported at room temperature to the lab and stored at 4 °C before use. Three samples were taken from Linfen (Shanxi, China), including expJ from a chicken stable, expY from a sheep stable and expT from a wheat field. The sample expM was taken from a horse stable in Hangzhou (Zhejiang, China). ExpJ, expY and expM were materials of stable soil with animal waste and ExpT was the soil in the wheat field; the samples were collected with a grid soil-sampling method composed of four parallel transects [40] in closed bags with the aseptic technique.

Deoxynivalenol (≥99.9%), (Triple Chemical Corp. Ltd., Guelph, ON, Canada), Acetonitrile (>99.9%), (Anhui Tiandi High Purity Organic Solvent Co., Ltd., Anqing, China), high-performance liquid chromatography (HPLC), and a Waters e2695 and Waters 2998 photodiode array detector (Waters Shanghai Corp., Ltd., Shanghai, China) were used.

Culture medium: NaHCO_3_ 0.06 g/L (Sinopharm Group Chemical Reagent Co., Ltd., Shanghai, China), KCl 0.01 g/L (Shanghai Lingfeng Chemical Reagent Co., Ltd., Shanghai, China), MgSO_4_·7H_2_O 0.12 g/L (Sinopharm Group Chemical Reagent Co., Ltd.), and CaCl_2_ 0.29 g/L (Chengdu Kelon Chemical Co., Ltd., Chengdu, China); pH 7.8 ± 0.2.

### 4.2. DON Degradation by Microbial Communities

DON solution at a concentration of 70 μg/mL was prepared with the culture medium in 4.1. First, 0.5 g of each soil sample was added into a sterile culture tube; then, 5 mL of 70 μg/mL DON solution was added, and the mixture was shaken at 30 °C and 130 rpm for 24 h, 48 h, 72 h and 96 h, respectively. Then, 1 mL of the solution was taken every 24 h and centrifuged at 5000× *g* rpm for 10 min; after being filtered through a 0.22 μm water nylon filter, DON was analyzed by HPLC. The remaining soil and supernatant (about 1 mL) were used for the microbial adsorption experiments. Monitored by HPLC analysis, DON in the soil and supernatants completely disappeared after 7 days. An amount of 0.2 mL of the supernatant was taken from each sample and was used for the microbial adsorption experiment. Another 0.2 mL of the supernatant was taken and sterilized as the control group.

For microbial adsorption analysis, the four unsterilized and the four sterilized supernatants (0.2 mL) were added to 0.8 mL of 100 μg/mL DON and cultivated at 30 °C for 96 h. The microbial adsorption effects were then analyzed by comparison between the DON concentration in the experiment and control groups. All sterilization in this study was carried out at 121 °C for 20 min.

### 4.3. Microbial Diversity Analysis of Mixed Flora in Samples

One of the three parallel groups of every soil sample after addition of DON solutions was selected and 0.5 g of each of the four soil samples was collected for high-throughput sequencing analysis of the microbial composition in the mixed flora in the soil (Azenta, Suzhou, China). The mixed flora in the four farm soils was labelled as expJ, expY, expT, and expM, respectively. After DON analysis as in 4.2, the supernatant was removed, and the residues were sent for microbial diversity analysis. The control groups of the mixed flora not treated with DON solution were labelled as controlJ, controlY, controlT, and controlM, respectively.

The genomic DNA of bacteria in the samples was extracted, the 16S rDNA variable region was amplified and the gene library was constructed. The multiple libraries were mixed and then sequenced by Illumina MiSeq to obtain the original data. Firstly, the raw data were de-linked and the low-quality data were filtered; then, the chimera sequences were removed to obtain the valid sequences for cluster analysis. One cluster named operational taxonomic units (OTU). The sequences were used for taxonomic analysis to obtain the species distribution information of each sample. Based on the OTU analysis results, a variety of alpha diversity indices were analyzed for each sample, and the species richness and evenness information of each sample were obtained. Based on taxonomic information, a statistical analysis of community structure at the genus level was performed. By calculating the Unifrac distance, constructing the UPGMA sample clustering tree, and drawing graphs such as PCoA, the differences in community structure between different samples or groups were visually displayed.

### 4.4. DON Analysis

DON was analyzed by HPLC as follows: the Shim-pack GWS C18 (5 μm, 250 × 4.6 mm) (Shimadzu Enterprise Management (China) Co., Ltd., Shanghai, China) was eluted with acetonitrile–water (20:80, *v*/*v*) at 0.8 mL/min and 35 °C, and the signal was detected at 218 nm with an injection volume of 10 μL.

The 10 mg/mL DON standard solution was prepared by dissolving 10 mg DON in 500 µL acetonitrile and diluted with 500 µL sterilized ultrapure water aseptically. After being thoroughly mixed, the solution was diluted to 0.5, 1, 5, 10, 50, and 100 μg/mL; after detection by HPLC, the linear regression equation of mass concentration and peak area was established, y = 4693.3x − 1439.5, and the correlation coefficient R^2^ = 0.9995, indicating that the mass concentration of DON had a good linearity with the corresponding DON concentration range of 0.5~100 μg/mL relation.

All LC-MS experiments were carried out on an Agilent 1290 ultra-performance liquid chromatography (UPLC) unit combined with an Agilent 6495 tandem triple quadrupole mass spectrometry spectrometer (Agilent, Santa Clara, CA, America). MS detection was carried out by using ESI in the positive mode under the following optimized parameters: scanning mode was full scanning mode with a mass range of *m*/*z* 100–800, corona current was 4 A, capillary voltage was 4500 V, nebulizer gas (N2) pressure was 20 psi, nebulizer gas (N2) flow rate was 14 L/min, sheath gas (N2) temperature was 200 °C, sheath gas (N2) flow rate was 11 L/min, and sheath gas (N2) temperature was 230 °C.

The UPLC analysis was performed on a Zorbax Eclipse XDB-C18 column (250 m × 4.6 mm, 5 μm) at a constant temperature of 35 °C, and achieved by an isometric elution with a mobile phase consisting of 0.1% formic acid in water methanol solution (V:V = 80:20) at a flow rate of 0.6 mL/min.

### 4.5. Calculation of DON Degradation Rate

The DON degradation rate was calculated according to the following equation:

(1) $$\mathrm{Degradation}\;\mathrm{rate}/\%=\frac{c_{0}-c}{c_{0}}\times 100\%$$

where *c*_0_ was the DON concentration added to the sample, and *c* was the DON concentration detected after treatment.

### 4.6. Statistical Analysis

The data obtained from the three parallel experiments are expressed as $\bar{x}$±s, and the statistical analyses were performed using SPSS software (17.0, Chicago, IL, USA) and, where appropriate, Tukey’s multiple comparison tests were used to determine if there was a significant difference between treatments.

## Figures and Tables

**Table 1:** Alpha diversity index.

Sample	Ace	Chao1	Shannon	Simpson	Goods_Coverage
controlJ	320.504	316.375	6.073	0.967	0.998
controlY	305.279	304.65	6.732	0.982	0.999
controlT	256.036	259.895	5.379	0.92	0.998
controlM	197.767	200.083	4.791	0.875	0.999
expJ	277.625	282.913	5.683	0.955	0.998
expY	281.069	279.955	6.575	0.982	0.999
expT	282.15	297.867	5.969	0.964	0.998
expM	144.405	184.25	4.886	0.938	0.999

## Data Availability

All data are included in this manuscript.
